# Lessons from Developing Multimedia Learning Materials for the Digital Generation

**DOI:** 10.1007/s43683-023-00110-w

**Published:** 2023-04-17

**Authors:** Jessica E. Herrmann, Susie Spielman, Ross Venook, Paul Yock, Lyn Denend

**Affiliations:** 1 Stanford University School of Medicine, 291 Campus Drive, Stanford, CA 94305 USA; 2 Stanford Byers Center for Biodesign, 318 Campus Drive, E100, Stanford, CA 94305 USA; 3 Stanford Bioengineering, 443 Via Ortega, Stanford, CA 94305 USA

**Keywords:** Online learning, Web-based learning materials, Teaching materials, Need-driven innovation, Health technology innovation

## Abstract

Recognizing that traditional textbooks on need-driven health technology innovation were increasingly misaligned with the needs of today’s undergraduate biomedical engineering students and the faculty who teach them, we initiated an effort to develop new learning materials for this audience. To guide our efforts, we conducted literature searches on best practices in the development of online content and engaging digital learners (primarily Gen-Z). We further held a series of discussions with biomedical engineering students and instructors at universities across the United States. This input led us to the development of a set of modular, online, multimedia learning materials specifically designed for the new generation of undergraduate learners. In this article, we present the key decisions that helped shape the project. We also share the results of feedback surveys and focus groups that shed light on how the materials have been preliminarily received. Finally, we reflect on challenges, opportunities, and lessons from this project that may be helpful to other initiatives focused on the creation of multimedia content for the digital generation.

## Challenge Statement

In the summer of 2019, a casual conversation in the hallway of Stanford Biodesign revealed an unmet educational need. An instructor of our graduate-level course on health technology innovation asked an instructor of the undergraduate bioengineering capstone which chapters they used in their class from *Biodesign: The Process of Innovating Medical Technologies,* an 800+ page textbook authored at Stanford Biodesign. The answer was a resounding “none.”

When we wrote the *Biodesign* textbook, our target audience was graduate and professional learners, and we intentionally sought to make the chapters comprehensive enough to be instructive in advancing real-world health technology innovation projects. However, this hallway discussion highlighted an unintended consequence: the depth of content was superfluous for undergraduate students. Further, the medium itself (a traditional textbook) was misaligned with the expectations digital learners have around fast, frequent interactions with content [1, 2]. This discussion led us to hypothesize that we could better address the educational needs of undergraduate instructors and their students—also referred to today as Gen-Z learners—with a more concise, mobile-ready “handbook” on the Biodesign approach to need-driven health technology innovation.

Before diving into development, we took a page from the innovation process we teach and decided to first research this unmet need, rather than relying solely on our own assumptions. We conducted literature searches on best practices in the development of online learning materials [3–5] and engaging Gen-Z learners [1]. In addition, we spoke with key stakeholders in the space—specifically, undergraduate students and instructors teaching design-oriented courses in biomedical engineering. Conversations with students from our bioengineering capstone course confirmed (1) their resistance to purchasing and using traditional textbooks; (2) their expectation of online accessibility (particularly as universities transitioned to distance learning during the COVID-19 pandemic [6]); and (3) their desire for short, targeted content. A subsequent focus group with instructors at the 2019 Biomedical Engineering Society (BMES) annual meeting provided insight into educators’ needs. In addition to concise materials that would resonate with their students, the instructors requested modular content they could use selectively to augment their existing lessons and materials. They also underscored the desire for videos, worksheets, templates, and other interactive tools that students could apply directly to their course-based projects in addition to traditional written content. Finally, they wanted dynamic materials that would be kept up to date as the health technology field evolved.

With this information in hand, we set out to develop a set of modular, online, multimedia learning materials specifically designed for the new generation of undergraduate learners.

## Novel Initiative

This initiative led to the creation of “A Student Guide to Biodesign,” available on an open-source basis at https://biodesignguide.stanford.edu/. The website currently includes 26 multimedia modules spanning the first two phases of the Biodesign process— “Identify” and “Invent” —as well as a section entitled “At Every Step” to address topics (e.g., teamwork, principled decision making) that should be top of mind throughout an innovation project. Each module, called a “toolkit,” includes a 5-10 page written brief, videos that underscore key take-aways, and one or more worksheets, templates, checklists, or resource listings to help students advance their projects.

In launching the effort, we made a series of decisions that were central to the direction of our project and relevant to initiatives focused on the creation of new learning materials for today’s undergraduate learners.

### Think Globally, Act Locally

Although we were inspired by the insightful feedback from instructors at other universities and committed to developing learning materials of value to the broader bioengineering educational community, we made an explicit decision to develop materials that would be directly and immediately useful to the classes we teach at Stanford. Our reason for this initial scope was to ensure that the project had a near-term “payback” on the time and resources we invested in the project. In addition, by beginning with our home institution, we could work directly with our colleagues and students to shape and pilot the materials before expanding access to other institutions.

### Begin with the End in Mind

Rather than planning the content serially, one or two modules at a time, we found it useful to conceptualize the entire Student Guide at the project outset. The benefits of this approach were that we could (1) estimate how much work lay ahead; (2) prevent informational gaps/overlap by pre-assigning topics to modules; and (3) more cohesively prioritize the order of module development.

### Define Clear Style/Content Guidelines

For any learning materials project, particularly those with multiple authors, it is essential to establish clear style and content guidelines. In this case, we outlined standards around word use, grammar, and punctuation, as well as content length, tone, voice, and presentation. See Table 1 for a sample of the decisions we made in advance of content creation. Importantly, we also selected an overall editor for the project, which is critical for ensuring consistency.

**Table 1:** Sample content/style guidelines for toolkits in “A Student Guide to Biodesign”

Category	Written Material Guidelines	Video Guidelines
Length	• Written briefs should not exceed 5-10 pages and/or take longer than 20-25 minutes to read	• Videos should ideally be ≤ 3 minutes, with 5 minutes as an absolute maximum
Voice	• We will address the reader in the second person (“you”) • We will use contractions (“you’re”) to give the materials a more conversational tone • Sentences should be short and simple • Avoid or define words that are not commonly used in conversation	• Primarily feature students sharing their experiences (rather than instructors or “experts”) • Keep style conversational
Presentation	• Use titles and subtitles for signposting throughout written content • Present content online in collapsible/expandable sections so students do not get overwhelmed by the amount of content on each page • Include multiple images, figures, and tables to add visual interest to each module • Provide a downloadable PDF option to enable offline reading	• Include a minimum of 1 video per toolkit, with 3-5 videos preferred • For consistency, open each video with a title slide and display a caption (name and affiliation) the first-time a speaker appears • Use B-roll throughout each clip to add visual interest and emphasize key take-aways • Close each video with a thank you to the speaker(s)
Other Content Guidelines	• Each toolkit should work as a standalone “lesson” so it can be directly linked to from a course syllabus or website • Liberally incorporate short, specific examples from student projects as case studies to underscore important points and/or tie concepts together • Include a minimum of at least 1 downloadable worksheet, template, or other learning resource in each toolkit to help students apply the skills to their projects • Include cross-references to other relevant toolkits and active links to additional resources to help with the discovery of related materials	• Each video should function as a standalone “lesson” and should not assume that students will have read the related written content • Videos will be filmed as interviews but the interviewer’s questions will be edited out to reduce the overall length of each clip (coach speakers to paraphrase the question in the opening of their response) • For multiple videos in a series, provide enough context in each video that students do not necessarily have to watch them in order

### Select a Platform

We had hoped to use an off-the-shelf learning management system (LMS) to publish the new materials. However, research into our options revealed that, while most LMSs allow for the creation of modular content, they often require modules to be presented in a “course” format such that students must complete each lesson sequentially. Given our desire for design flexibility and input from instructors at other universities about their plans for incorporating the modular content, we decided to build our own website using a standard content management system (CMS). We selected WordPress for its prevalence, support across multiple browsers/devices, straightforward backend, and extensive add-on features. Understanding the capabilities of the platform early in the content development cycle enabled us to optimize our approach based on the opportunities and constraints of the technology.

### Decide on an Access Strategy

Another important decision was whether to restrict and/or charge for access to the content. Given the substantial investment of time and energy to develop the Student Guide, and the need to maintain materials into the future, there was some rationale for devising a subscription model to help cover upfront and continuing costs. However, since accessibility to students was a primary driver of the project, we decided that open access would best enable learners everywhere to benefit from the materials.

### Pilot, Learn, Improve, Repeat

Rather than releasing all content in a single launch, we staged the development effort to enable us to collect and act on feedback throughout the project. We initially created 11 toolkits, which we “mocked up” and made available online to early users (August 2020). We then recruited student volunteers from past Biodesign classes to review the materials, offering modest incentives ($25 gift cards) for each toolkit evaluation. In parallel, we shared the materials with the instructors who had participated in the focus group at BMES. The resulting feedback on content length, depth, and tone, as well as website design, proved invaluable in improving the materials for the next cycle. Roughly one year later (August 2021), we launched http://biodesignguide.stanford.edu with a total of 20 multimedia toolkits and, again, invested in gathering feedback from students and instructors, this time on a more widespread basis, to test and direct our work (see below).

## Reflection

We initially began using the Student Guide materials in three introductory courses at our institution and found them to be immediately valuable as an alternative to the textbook. Typically, specific toolkits are assigned ahead of class as pre-work, with 5-question quizzes used to reinforce key take-aways. Lecture content builds on the written toolkit content (rather than repeating it). We periodically show videos from the Student Guide of students applying key concepts to their projects, either to introduce topics or to reinforce their application. Worksheets and templates are assigned as homework and/or used during in-class work time.

To help us reflect on this project—and in keeping with our “pilot, learn, improve, repeat” approach—we conducted surveys and focus groups after the 20 toolkits in the Student Guide had been available for most of the 2021-22 academic year. One advantage of online learning materials, distinct from a traditional textbook, is the ability to gather basic information about individual users. In our case, we deployed a brief registration form to collect user names and email addresses, as well as high-level information regarding affiliation, location, and motivation for accessing the website. This form is presented to all first-time visitors. We leveraged the contact information to survey users, on a voluntary, opt-in basis, regarding their experience with the materials. We received responses from 47 students and 39 instructors (response rates 9.6% and 21.3%, respectively, of the sample surveyed). One question on the survey was whether respondents would be willing to participate in a focus group (see Table 2 for the other survey questions). Of those who volunteered, 22 undergraduate students (~90% from Stanford) and five instructors (all from different institutions) participated in subsequent Zoom discussions.

**Table 2:** Survey Questions

*Both Students and Instructors*
1. What was your primary reason for using the materials? 2. Overall, how helpful did you find the materials for learning about need-driven health technology innovation? 3. Overall, how easy were the materials to access, navigate, and use? 4. Compared to a traditional textbook, how engaging did you find the materials? 5. How would you rate content depth (comprehensiveness, level of detail)? 6. How would you rate content length (amount of time needed to read/watch content)? 7. How would you rate the content tone (readability, understandability)? 8. What did you like most about the Student Guide to Biodesign? 9. What did you like least about the Student Guide to Biodesign? 10. Is there anything else you’d like to tell us to help us improve the Student Guide to Biodesign? 11. Would you be willing to participate in a 45 minute focus group to provide further feedback?
*Student-Specific*
1. Once registered, did you access the materials on the Student Guide to Biodesign website? If no, why didn’t you access or use the materials? 2. Compared to a traditional textbook, do you think you accessed/used the Student Guide materials more or less frequently? 3. What type(s) of content did you find most valuable? (choose all that apply) 4. What type(s) of content did you access/use most often? (1=most to 5=least) 5. How did you typically use the Student Guide materials? (choose all that apply) 6. How long did you typically spend on the website during a normal visit?
*Instructor-Specific*
1. Once registered, did you access and use the multimedia learning materials on the Student Guide to Biodesign website? 2. How did you assign/utilize the resources in the Student Guide to Biodesign? (choose all that apply) 3. In your course/program, which Identify toolkits did you use/assign? (check all that apply) 4. In your course/program, which Invent toolkits did you use/assign? (check all that apply) 5. In your course/program, which And Beyond toolkits did you use/assign? (check all that apply) 6. In your course/program, did you use the additional resources such as videos, downloadable templates, worksheets, briefs, etc.? 7. To what extent (if at all) did you see a difference in student performance after using materials from the Student Guide to Biodesign? 8. Did your students provide feedback on their experience using the Student Guide to Biodesign?

**Fig. 1:**
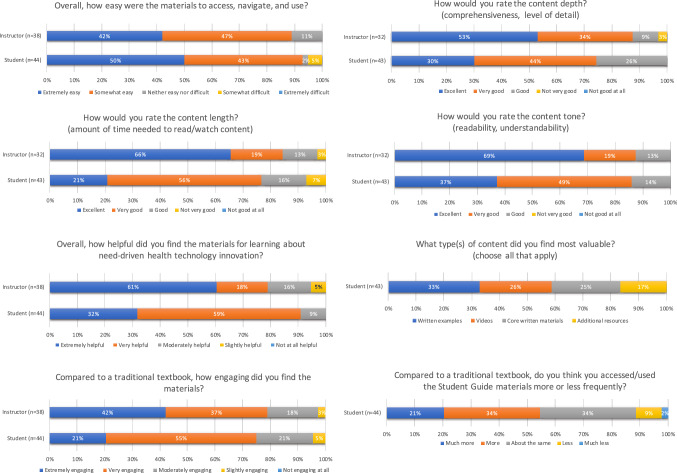
Student and Instructor Survey Feedback

Our main goal during feedback collection was to determine, before releasing additional materials, whether the content aligned with user needs in terms of its perceived value, usability, targeting, and engagement. The survey feedback (see Fig. 1) indicated that students and instructors both found the Student Guide beneficial for learning about need-driven health technology innovation. The input confirmed that the online materials were relatively easy to access, use, and navigate. Both groups indicated that content length, depth, and tone were generally on target. A majority of students and instructors reported that the materials were engaging, with many student respondents indicating that they accessed the Student Guide “more” or “much more” than a traditional textbook.

By conducting the surveys and focus groups in combination, we could seek clarification from focus group participants on survey results. For example, by speaking with students about content length, we learned that they preferred toolkits without large or uninterrupted blocks of text. This underscored the importance of not only reducing content, but also visually interrupting text with interactive features (e.g., graphics, videos, collapsible sections). Both user groups found the case studies, which were presented in written and video form, particularly beneficial for learning. However, we were surprised that the worksheets, templates, and checklists were utilized less than anticipated. Through our focus groups, we learned that students and instructors alike often overlooked these materials due to how they were presented online. This clarification enabled us to make multiple website changes, including the creation of a resource library where these supplemental materials can be more easily accessed.

Beyond the surveys and focus group, we identified at least one unexpected opportunity and two ongoing challenges that require additional attention.

Encouragingly, we discovered that the Student Guide is useful for introductory learners beyond undergraduate students. We initially started using select toolkits in our graduate course, then expanded into our faculty fellowship and executive education program. Rather than finding the materials rudimentary, these more advanced learners shared anecdotal input with us that they appreciate their brevity and clarity as they initially familiarize themselves with Biodesign concepts. Then, as their understanding deepens and their projects advance, they transition to more in-depth content in the *Biodesign* textbook. This blended model, with the Student Guide serving as an “on ramp” to the more comprehensive materials, has proven effective.

In terms of challenges, we are acutely aware of the ongoing resource requirements associated with online learning materials. Unlike a traditional textbook that is considered complete once published, online materials require a sustained investment. Dedicated personnel are needed to keep content up to date, expand materials and modify the user interface in response to incoming feedback, validate new and substantially edited content with relevant subject matter experts, and manage website maintenance. This creates an ongoing need for staffing and funding, which can be challenging in an academic environment.

Additionally, without a traditional publisher to assist with promotion of the materials, we have encountered challenges in driving adoption of the Student Guide. One year after the launch of https://biodesignguide.stanford.edu/ we had just over 1,200 registered users (54% students/learners, 21% professors/instructors/trainers, 19% industry/government professionals, 6% other). Nearly one-quarter of these individuals are based at Stanford. A total of 291 distinct organizations (74% academic institutions, 24% companies) have used the content in some capacity but, based on the number of registered users in each organization, this figure largely reflects individual use rather than widespread adoption at each organization. While these figures reflect reasonable uptake given the newness of the initiative, we believe that many more learners could benefit from the materials. To date, we have advertised the Student Guide on the Stanford Biodesign website and social media channels, including Facebook, LinkedIn, and Twitter. Moving forward, we intend to diversify our promotion efforts with the hope of expanding the Student Guide’s reach.

Overall, this project has substantially advanced our approach to developing educational materials for digital learners, and the results suggest that we are heading in the right direction. Next, we must consider the implications on developing more in-depth and comprehensive resources—at the level of the *Biodesign* textbook—for the next generation of students.

## Data Availability

Not applicable.
